# Prevalence, characteristics, and management of childhood functional abdominal pain in general practice

**DOI:** 10.3109/02813432.2013.844405

**Published:** 2013-12

**Authors:** Leo A. A. Spee, Yvonne Lisman-Van Leeuwen, Marc A. Benninga, Sita M. A. Bierma-Zeinstra, Marjolein Y. Berger

**Affiliations:** ^1^Department of General Practice, Erasmus MC University Medical Centre, Rotterdam, the Netherlands; ^2^Department of General Practice, University of Groningen, University Medical Center Groningen, the Netherlands; ^3^Department of Pediatric Gastroenterology, Emma Children's Hospital/ Academic Medical Center, Amsterdam, the Netherlands

**Keywords:** Abdominal pain, adolescent, child, diagnosis, family practice, general practice, the Netherlands

## Abstract

**Objective:**

To (i) describe the proportion of children presenting with abdominal pain diagnosed by the GP as functional abdominal pain (GPFAP); (ii) evaluate the association between patient and disease characteristics and GPFAP; (iii) describe diagnostic management by the GP in children presenting with abdominal pain, and (iv) evaluate whether children with GPFAP fulfill diagnostic criteria for functional abdominal pain (FAP) as described in current literature: chronic abdominal pain (CAP) and the Rome III criteria (PRC-III) for abdominal pain-related functional gastrointestinal disorders (FGID).

**Design:**

Cross-sectional study.

**Setting:**

General practices in the Netherlands.

**Subjects:**

305 children aged 4–17 years consulting for abdominal pain.

**Main outcome measures:**

GPFAP, CAP, FGIDs.

**Results:**

89.2% of children were diagnosed with GPFAP. Headaches and bloating were positively associated with GPFAP whereas fever and > 3 red flag symptoms were inversely associated. Additional diagnostic tests were performed in 26.8% of children. Less than 50% of all children with GPFAP fulfilled criteria for CAP and FGIDs; in 47.9% of patients the duration of symptoms at presentation was less than three months.

**Conclusions:**

In almost 90% of children included in this study the GP suspected no organic cause for the abdominal pain. GPs diagnose FAP in children without alarm symptoms and order diagnostic testing in one out of four children presenting with abdominal pain. No difference was found in GPs’ management between children with a diagnosis of GPFAP and other diagnoses. Only about half of the children with a GP diagnosis of FAP fulfilled time-criteria of FAP as defined in the literature.

Although children with functional abdominal pain (FAP) are mainly managed in primary care, not much is known about FAP in this setting. This study showed that:

- In almost 90% of children presenting with abdominal pain included in this study, the general practitioner (GP) suspects no organic cause.
- GPs diagnose FAP in children without red flag symptoms and order diagnostic testing in one out of four children presenting with abdominal pain.
- Only 50% of children with a diagnosis of FAP by their GP fulfill the time-criteria for FAP as defined in the literature.

## Background

Abdominal pain accounts for 5% of childhood consultations in general practice [1]. It has a major impact on the child's well-being and the healthcare system [2,3]. Usually, this complaint is not associated with organic disease and is labeled as functional abdominal pain (FAP).

There are different approaches for defining childhood FAP. In 1958, Apley described recurrent abdominal pain as ≥ 3 bouts of pain, severe enough to affect activities, over a period of at least three months [4]. Von Baeyer added criteria for impact on daily functioning, and called it chronic abdominal pain (CAP) [5]. The “Pediatric Rome Criteria III” (PRC-III) classified abdominal pain-related functional gastrointestinal disorders (FGIDs) using a symptom-based approach [6]. We assume that children suspected of FAP in general practice have comparable characteristics to children receiving a diagnosis of FAP in other settings.

To fulfill these definitions organic diseases need to be excluded. The extent of diagnostic testing is left to the decision of the clinician. Wanting to avoid unnecessary medical testing in children presents GPs with the difficult task of reassuring themselves and their patients that no significant causes are missed. To date, no symptoms, signs, or tests have been reported to help discriminate between organic and functional abdominal pain [7]. We therefore assume that in general practice, diagnostic testing in children presenting with abdominal pain will be directed towards ruling in or out organic disease, rather than towards diagnosing FAP.

The present study investigates the proportion of FAP in children presenting with abdominal pain in general practice according to different definitions, evaluates the association between patient and disease characteristics and a GP diagnosis of FAP (GPFAP), and describes GPs’ diagnostic management.

## Material and methods

### Design and setting

We performed a cross-sectional analysis of baseline data of the HONEUR abdominal pain cohort. Fifty-three GPs, together comprising a population of 16.000, children aged 4–17 years, were recruited in Rotterdam, a multicultural city, and its rural surroundings. GPs recruited consecutive children consulting for abdominal pain during a two-year period. A child was eligible if the consultation was not preceded by a consultation for this complaint in the previous three months. After written informed consent, a research nurse visited the children within one week and collected data. Included children were younger than eligible non-included children (mean 8.5 versus 9.2 years); fewer children diagnosed with “gastroenteritis” and more with “generalized abdominal pain” were included. Therefore, our cohort represents young school-aged children consulting their GP with a new episode of abdominal pain not obviously related to gastroenteritis [8].

### Selection of determinants

We evaluated the association between GPFAP and characteristics reported to be associated with FAP in a systematic review [7]. In addition we evaluated red flag symptoms reported to be associated with organic disease in (inter)national guidelines [7,9,10]. Demographic data, additional symptoms, comorbidity, and family history were recorded on structured questionnaires. For assessment of somatization, we used the somatic syndrome scale of the Child Behavior Checklist [11–13]. Pain intensity was determined on an 11-point numerical rating scale (NRS) in children aged 8–17 years; and on a six-point pain faces scale for children < 8 years [14]. The GP's management was recorded in 16 structured and one open question. Description of the data collection is described in detail elsewhere [8].

### Primary outcome GPFAP

GPs coded consultations according to the International Classification of Primary Care (ICPC) [15]. These codes were extracted from the medical records three months after inclusion. Of the children that consulted the GP more than once for abdominal pain in this three-month period, in 19 children the initial code was changed. For this analysis the last given ICPC code was used. We considered the following diagnoses as GPFAP: “abdominal pain, general” (D01), “epigastric pain” (D02), “abdominal pain, localized other” (D06), “constipation” (D12) and “irritable bowel syndrome (IBS)” (D93). A study in the Netherlands showed an agreement for “generalized abdominal pain” between GPs and experts of 85% [16].

### Definitions of FAP as defined in the literature

1. CAP: The occurrence of abdominal pain at least once each month in the past three months, severe enough to stay home from school, terminate or avoid play, take medication for the pain, or to be rated as moderate to severe (≥ 3/10 on the NRS) [5].
2. FGIDs: IBS, functional dyspepsia, functional abdominal pain, and functional abdominal pain syndrome [6]. The PRC-III define a time period of two months in which the symptoms had to occur at least once a week; however, we used the timeline as proposed by Von Baeyer (Supplementary Appendix I available online at http://informahealthcare.com/doi/abs/10.3109/02813432.2013.844405).

### Statistical analyses

Data are presented as means with standard deviations (SD) or percentages of the number of patients responding per item. Factors associated with GPFAP were identified by logistic regression analyses adjusted for age. The association between GP management and GPFAP was evaluated by logistic regression analyses and adjusted for potentially relevant confounders identified in the bivariate analysis. Results are expressed as odds ratios (OR) and 95% confidence intervals (95%CI). Analyses were performed using SPSS version 17.0.

## Results

### Study sample

In total 305 of 348 invited children (87.6%) gave informed consent and participated in the study. Mean age was 8.3 years and 62.0% were female. Eight consultations were not given an ICPC code and it was not possible to determine the diagnosis based on information in the medical records. In 297 children a diagnosis was available, of which 265 were diagnosed as GPFAP (89.2%) (Table I).

**Table I.** Univariate analysis of patient and pain characteristics and diagnosis of functional abdominal pain by the general practitioner (GPFAP).

Characteristics	GPFAP n = 265	Organic n = 32	OR (95%CI) adjusted for age
Demographics: Age, mean (± SD) Sex (% female) Factors associated with FAP, n (%): Belching Flatulence Bloated feeling Dyspepsia Headache on a regular basis Somatization Family history of functional GI complaints Family history of regular headaches Red flag symptoms, n (%) > 3 red flag symptoms Vomiting Wake up at night due to abdominal pain Pain urinating Fever Underweight Blood on stool Intra-abdominal comorbidity Abdominal surgery in history Gastroenteritis in previous year Urinary tract infection in previous year	8.55 (2.99) 162 (61.1) 42 (15.8) 85 (32.1) 91 (34.6) 54 (20.4) 98 (37.0) 51 (19.2) 81 (31.3) 116 (44.4) 8 (3.0) 39 (14.7) 104 (39.2) 17 (6.4) 36 (13.6) 22 (8.3) 4 (1.5) 168 (63.4) 9 (3.4) 157 (59.2) 24 (9.1)	6.27 (1.78) 21 (65.6) 7 (21.9) 8 (25.0) 5 (15.6) 3 (9.4) 6 (18.8) 2 (6.3) 9 (28.1) 15 (46.9) 6 (18.8) 9 (28.1) 14 (43.8) 2 (6.3) 13 (40.6) 2 (6.3) 2 (6.3) 26 (81.3) 2 (6.3) 24 (75.0) 8 (25.0)	1.45 (1.20–1.75)* 0.82 (0.38–1.78) 0.67 (0.27–1.66) 1.42 (0.61–3.28) 2.86 (1.07–7.67)* 2.47 (0.73–8.43) 2.54 (1.01–6.39)* 3.58 (0.83–15.45)** 1.16 (0.52–2.63) 0.91 (0.43–1.89) 0.20 (0.06–0.65)* 0.44 (0.20–1.02)** 0.83 (0.40–1.74) 1.03 (0.23–4.67) 0.23 (0.10–0.51)* 1.36 (0.30–6.06) 0.23 (0.04–1.32)** 0.40 (0.16–1.01)** 0.53 (0.11–2.56) 0.49 (0.21–1.12)** 0.30 (0.12–0.74)*

Notes: *p ≤ 0.05; **p < 0.10.

### Characteristics associated with GPFAP

The chance of a diagnosis of GPFAP increased with increasing age. Headaches and bloating were associated with GPFAP. Fever and a UTI in the past year were negatively associated with GPFAP; vomiting, blood on stool, and intra-abdominal comorbidity showed a trend towards an inverse association. Having > 3 red flag symptoms showed a significant inverse association with GPFAP (see Table I).

### Management by GP

Additional diagnostic testing was performed in 26.3% of children; 10.1% of children were referred to specialist care. No differences were observed in diagnostic management by the GP between children with and without GPFAP and between children with or without > 3 red flag symptoms. The association between GP management and GPFAP remained insignificant when adjusting for potentially relevant confounders (Table II). Of 265 children with GPFAP the GP ordered blood sampling in 23.0% of children, abdominal X-rays or ultrasonography in 8.3%, and both in 4.5%.

**Table II.** Multivariate analysis of management by general practitioner (GP) and a diagnosis of functional abdominal pain by the GP (GPFAP).

			OR (95%CI)	
Management and diagnosis by GP	GPFAP n = 265	Organic n = 32	Unadjusted	Adjusted for potential confounders*
Diagnosis GP, ICPC code: Generalized abdominal pain (D01) Gastric pain (D02) Localized abdominal pain (D06) Constipation (D12) Gastroenteritis (D11/D70/D73) Urinary tract infection (U71) Appendicitis (D88) Other FGIDs according to the PRC-III: FGIDs, general Functional dyspepsia Irritable bowel syndrome Functional abdominal pain Functional abdominal pain syndrome Additional diagnostic tests: Abdominal X-ray/Ultrasonography Blood sampling Referral to specialist care	174 (65.7) 2 (0.8) 57 (21.5) 31 (11.7) – – – 1 (0.4) 130 (50.6) 10 (7.7) 50 (38.5) 70 (53.8) 67 (51.5) 71 (26.8) 22 (8.3) 61 (23.0) 27 (10.2)	– – – – 17 (53.1) 5 (15.6) 1 (3.1) 9 (28.1) 7 (21.9) 1 (3.1) 6 (18.8) 3 (9.4)	1.31 (0.54–3.16) 1.10 (0.31–3.84)	0.75 (0.28–1.98) 1.11 (0.30–4.19)

Notes: *Age, > 3 red flag symptoms, and intra-abdominal comorbidity.

### Relation between definitions

Of 265 children with GPFAP, 130 (50.6%) fulfilled FGID criteria: 53.8% fulfilled criteria for FAP, 38.5% for IBS, and 7.7% for functional dyspepsia (see Table II). All children with GPFAP not fulfilling FGID criteria lacked the time criterion of three months.

Of children with GPFAP, 47.9% fulfilled criteria for CAP; of children with GPFAP not fulfilling CAP criteria 92.0% lacked the time criterion.

## Discussion

The present study showed that in 90% of children presenting with abdominal pain, the GP suspected FAP. Older age and the coexistence of headaches and bloating were associated with GPFAP. Fever, a UTI in the past year and having > 3 red flag symptoms were inversely associated with GPFAP. The GP ordered additional testing in one out of four children. No differences in GPs’ management were observed between children with and without GPFAP. Only about 50% of children with GPFAP fulfilled the criteria for CAP or an FGID. This discrepancy was due to the shorter duration of complaints than the 3 months in the respective definitions.

In 90% of children the GP suspects FAP; this is consistent with the findings of Apley who found somatic causes in 6–8% of children with recurrent abdominal pain in population-based studies [17]. Recent studies found organic abnormalities in 45–88% [18,19]; however, these studies were performed in specialist-care settings and selection of patients and excessive testing may have yielded higher proportions of organic abnormalities. Furthermore, abnormal findings are not necessarily causally related to abdominal pain.

The observation that age was significantly associated with GPFAP is in accordance with the findings of others that the prevalence of chronic pain increases with age [20]. Headache and bloating were positively associated with GPFAP. Headache is, together with limb and abdominal pain, the most frequently reported functional complaint in children [21]. Bloating is a common, not well-defined symptom in adults related to IBS [22]. Somatization and a family history of GI complaints have been found by others to be associated with FAP. Although somatization showed a tendency toward a statistically significant association with GPFAP (p = 0.08) we could not fully confirm these findings, possibly due to a lack of power. Given that children with GPFAP have a shorter duration of symptoms compared with children with FAP in specialist care, our finding may indicate that these characteristics are related to the duration of symptoms rather than to symptoms of FAP.

The finding that red flag symptoms were inversely associated with GPFAP is in accordance with (inter)national guidelines in which red flag symptoms are associated with a higher risk of organic disease [7,23]. These results support our hypothesis that the diagnostic management of children with abdominal pain is directed towards the in- or exclusion of organic causes.

According to guidelines, organic disease needs to be excluded before a diagnosis of FAP can be made. However, in a population with a small prior probability of organic disease, the risk of false positive findings will be relatively high. Testing will therefore not be cost-efficient and introduces unnecessary parental worries. From this perspective additional testing in one out of four children seems high.

As the point of entry to healthcare, many children will visit the GP with short-term symptoms. Together with the low prior probability of somatic pathology, this enforces GPs to consider FAP in an early stage. Given the high percentage of GPFAP in children lacking chronicity, the GP seems confident in giving a symptom-diagnosis after exclusion of organic disease. He might presume the patient will return in the case of persistence of complaints.

Compared with others, we found a higher proportion of children fulfilling the PRC-III for FAP [24,25], which might indicate subgroup misclassification. A possible explanation is that we were not able to assess abdominal migraine as its criteria were considerably revised in the updated criteria. Furthermore, we did not use the sub-classification of functional constipation as this is not considered an abdominal pain-related FGID. Therefore, children otherwise fulfilling criteria for abdominal migraine or functional constipation were “misclassified” as FAP.

### Study limitations

First, the presence of patient determinants was assessed using standardized questionnaires and we are not sure whether the GP used these determinants in his diagnostic reasoning. However, the red flag symptoms were selected from (inter)national guidelines and we may therefore reasonably assume the GPs did include them in their diagnostic reasoning.

Second, we were not able to use the “Rome III Diagnostic Questionnaire for Pediatric FGIDs” as this study was ongoing at the time of its publication; nevertheless, we used equivalent questions used for the assessment of FGIDs (Supplementary Appendix I available online at http://informahealthcare. com/doi/abs/10.3109/02813432.2013.844405). This might have led to subgroup misclassification as was pointed out in the discussion section.

Third, we used different criteria for required symptom duration than proposed by the PRC-III. Although the required duration of two months includes more children fulfilling FGID criteria, the less stringent criterion for symptom frequency is more likely to have led to an overestimation of the prevalence of FGIDs in our cohort.

Fourth, the power of our study was lower than the expected 80% [8]. Given a prevalence of GPFAP of 90% and a distribution of determinants between children with and without GPFAP of 90% versus 10%, we had a power of 72% to detect an OR of 2.5 with an α of 0.05. There is a chance of 28% that our results were found by chance. We feel, however, that a loss of power of 8% is not enough to influence our conclusions.

### Clinical implications and future research

Additional diagnostic testing is performed in one out of four children with abdominal pain. However, we found heterogeneity in the kind of tests asked for, and there is a lack of evidence for their diagnostic value in primary care. Therefore, studies on the diagnostic value of additional diagnostic tests in children with abdominal pain in primary care are essential. To our knowledge these studies are lacking, which might be explained by the fact that evaluating diagnostic tests in a population with low prior probabilities of disease is a methodological challenge.

In a prior Dutch cohort of children with abdominal pain, it was found that the consultation rate after first presentation of abdominal pain was 21.9% [1], indicating that active follow-up of children with FAP is not common practice. Instead of managing FAP by focusing on the exclusion of organic disease, the GP could more often use active follow-up to monitor the course of the complaints. Our finding that only half of the children with GPFAP had chronic complaints, whereas FAP in referred children seems a chronic condition, makes follow-up even more warranted. To date, there is a lack of knowledge on the prognosis of FAP and its determinants emphasizing that studies on this topic are highly recommended.

## Conclusion

In our study GPs suspected FAP in almost 90% of children visiting with abdominal pain. Only about 50% of children with a diagnosis of GPFAP fulfilled the criteria for CAP or an FGID due to a short duration of their complaints at presentation. GPs suspect FAP in children without red flag symptoms and order additional tests in one out of four. It remains inconclusive whether a child suspected of FAP in general practice has comparable characteristics to a child with FAP diagnosed in other settings. For better understanding of FAP and its prognosis in primary care further studies in this setting are needed.
