# Supplementary material for: Prevalence, characteristics, and management of childhood functional abdominal pain in general practice
Source: Scand J Prim Health Care. 2013 Dec;31(4):197–202. doi: 10.3109/02813432.2013.844405 (PMC3860295; doi:10.3109/02813432.2013.844405)
Supplement: Supplementary file 1 [file pri-31-197-s001.pdf]

Supplementary material for Spee L. A. A, et al. Prevalence, characteristics, and management of childhood functional abdominal pain in general practice. Scand J Prim Health Care 2013;31:197–202.

Supplementary Appendix 1. Pediatric Rome III criteria (PRC-III, left) and the criteria used in our cohort to assess the PRC-III criteria (right).

PRC-III	Assessment of the PRC-III, using validated questionnaires
At least *once per week during at least 2 months* before diagnosis, and no evidence of an inflammatory, anatomic, metabolic, or neoplastic process that explains the subject's symptoms	At least *once a month during at least 3 consecutive months*, and no evidence of an inflammatory, anatomic, metabolic, or neoplastic process that explains the subject's symptoms
**Functional dyspepsia (FD)**	
Must include *all* of the following:<br>1. Persistent or recurrent pain or discomfort centered in the upper abdomen (above the umbilicus)<br>2. Not relieved by defecation, or associated with the onset of a change in stool frequency or stool form (i.e. not IBS)	1. Where is pain localized most of the time?<br>2. a. Is the pain relieved by defecation?<br>b. Was the onset of pain associated with a change in stool frequency?<br>c. Was the onset of pain associated with a change in stool form?
**Irritable bowel syndrome (IBS)**	
Must include *all* of the following:<br>1. Abdominal discomfort or pain associated with two or more of the following at least 25% of the time:<br>a. Improved with defecation<br>b. Onset associated with a change in frequency of stool<br>c. Onset associated with a change in form (appearance) of stool	1. a. Is the pain relieved by defecation?<br>b. Was the onset of pain associated with a change in stool frequency?<br>c. Was the onset of pain associated with a change in stool form?
**Functional abdominal pain (FAP)**	
Must include *all* of the following:<br>1. Episodic or continuous abdominal pain<br>2. Insufficient criteria for other FGIDs	1. Fulfilling the time criterion by Von Baeyer<br>2. Not fulfilling criteria for functional dyspepsia or IBS
**Functional abdominal pain syndrome (FAPS)**	
Must include Childhood Abdominal Pain at least 25% of the time and *one or more* of the following:<br>1. Some loss of daily functioning	Fulfilling criteria for Childhood Functional Abdominal Pain and<br>1. The pain had an impact on the child's daily functioning: staying home from school, terminating or avoiding play, taking medication for pain or rating the pain as moderate to severe (≥ 4 on an 11-point scale).<br>2. Having somatization problems according to the CBCL somatic syndrome scale.
